# Physicochemical Characterization and Paper Spray Mass Spectrometry Analysis of *Myrciaria Floribunda* (H. West ex Willd.) O. Berg Accessions

**DOI:** 10.3390/molecules26237206

**Published:** 2021-11-27

**Authors:** Yesenia Mendoza García, Ana Luiza Coeli Cruz Ramos, Afonso Henrique de Oliveira Júnior, Ana Cardoso Clemente Filha Ferreira de Paula, Angelita Cristine de Melo, Moacir Alves Andrino, Mauro Ramalho Silva, Rodinei Augusti, Raquel Linhares Bello de Araújo, Eurico Eduardo Pinto de Lemos, Júlio Onésio Ferreira Melo

**Affiliations:** 1 Centro de Ciências Agrárias, Campus A. C. Simões, Universidade Federal de Alagoas, Rio Largo 57072-970, Brazil; jenny_thesiba@hotmail.com (Y.M.G.); eurico@ceca.ufal.br (E.E.P.d.L.); 2 Departamento de Alimentos, Faculdade de Farmácia, Campus Belo Horizonte, Universidade Federal de Minas Gerais, Belo Horizonte 31270-901, Brazil; analuizacoeli@gmail.com (A.L.C.C.R.); raquel@bromatologiaufmg.com.br (R.L.B.d.A.); 3 Departamento de Ciências Exatas e Biológicas, Campus Sete Lagoas, Universidade Federal de São João Del-Rei, Sete Lagoas 36307-352, Brazil; afonsohoj@gmail.com (A.H.d.O.J.); angelitamelo@ufsj.edu.br (A.C.d.M.); 4 Departamento de Ciências Agrárias, Instituto Federal de Educação, Ciência e Tecnologia de Minas Gerais, Campus Bambuí, Bambuí 38900-000, Brazil; ana.paula@ifmg.edu.br (A.C.C.F.F.d.P.); moacir.andrino@hotmail.com (M.A.A.); 5 Departamento de Nutrição, Pontifícia Universidade Católica de Minas Gerais, Campus Barreiro, Belo Horizonte 30640-070, Brazil; mauroramalhosilva@yahoo.com.br; 6 Departamento de Química, Campus Belo Horizonte, Universidade Federal de Minas Gerais, Belo Horizonte 35702-031, Brazil; augusti.rodinei@gmail.com

**Keywords:** Cambuí, Brazilian flora, physicochemical characterization, phenolic compounds, flavonoids

## Abstract

*Myrciaria floribunda*, also known as rumberry, is a tree native to the Brazilian Atlantic Forest, where its fruits have the potential for commercial use. This study evaluates the antioxidant potential, determines the phytochemical profile, and chemically characterizes the rumberry fruit. Accessions were sampled from the Rumberry Active Germplasm Bank of the Federal University of Alagoas, Brazil. Physical characteristics, chemical characteristics, and phenolic compound content were analyzed. Chemical profile characterization was carried out using PSMS. Accessions had an average weight of 0.86 g. Accession AC137 presented a higher pulp yield (1.12 g). AC132 and AC156 had larger fruits, AC137 showed greater firmness (5.93 N), and AC160 had a higher and total phenolic content ratio (279.01 ± 11.11). Orange-colored accessions scored higher in evaluated parameters, except for AC160 for phenolic content. Thirty-two compounds were identified on positive ionization mode and 42 compounds on negative ionization mode using PSMS. Flavonoids, followed by the derivatives of benzoic acid, sugars, and phenylpropanoids, were the most prominent. Myricitrin, quercitrin, and catechin stand out as flavonoids that have been reported in previous studies with antioxidant and antimicrobial properties, in addition to health and therapeutical benefits, demonstrating the potential of the rumberry fruit.

## 1. Introduction

*Myrtaceae* is one of the most representative families of Angiosperms, distributed throughout Australia, South America, Tropical America, and Southeast Asia, though mainly found in tropical and subtropical regions. In Brazil, the family is considered one of the most economically important, occupying the eighth position regarding the number of species. In the country’s Northeast region, specifically in the state of Alagoas, there are records of over 1800 species of angiosperms, 11 of which are endemic to the region [1].

Brazil stands out as one of the largest producers of fresh fruit. It has a sizeable territorial extension with favorable climatic conditions for producing native and exotic fruit species, among them the rumberry, known as cambuí natively. Many native species are still unknown to the general public but have great economic potential for fresh or processed fruit marketing. Among the native fruits with great potential for industrialization is the rumberry (*Myrciaria floribunda*). The Brazilian name “cambuí” is of indigenous origin, meaning “shedding leaf”, a designation given to specimens belonging to the genera *Myrcia* and *Myrciaria* [1,2].

The rumberry tree is a fruit tree that grows naturally in a wide range of environments, distributed throughout Northeast and Northern South America and Central America. In Brazil, this species can be found in the Atlantic Forest biome in the Northeastern region, more abundantly in the states of Alagoas and Sergipe [3]. The species has several botanical synonyms as *Eugenia floribunda* H. West ex Willd., *Eugenia oneillii* Lundell, *Eugenia protracta* Steud., *Eugenia salzmannii* Benth., *Myrciaria mexicana* Lundell, *Myrciaria oneillii* (Lundell) I.M. Johnst, *Myrciaria protracta* (Steud.) O. Berg, *Myrciaria salzmannii* (Benth.) O. Berg, *Myrciaria uliginosa* O. Berg, *Myrciaria verticillata* O. Berg, *Myrciaria ciliolata* (Cambess) O. Berg, and *Myrciaria tenuiramis* O. Berg [1,2,3].

Rumbery’s trees are shrub or tree that has potential for ornamental use. Its fruits are suited for human consumption in natura or processed in the form of beverages (liqueurs, juices, soft drinks), jams (jellies and ice creams), frozen, dehydrated, or freeze-dried. Fruits are small globular berries of up to 13 mm in diameter varying in color, with yellow, orange, red, or purple coloration when ripe, depending on the plant. The peel is relatively thin, and the fruit has excellent pulp yield, characterized as juicy with a sweet, astringent flavor and a much-appreciated characteristic aroma and taste regionally [4,5].

Rumberry fruits present physical and physicochemical characteristics according to maturity. It is rich in sugars, vitamin C, bioactive compounds such as flavonoids, carotenoids, and phenolic compounds, demonstrating an excellent antioxidant potential [6]. Many medicinal properties are attributed to the chemical composition of the essential oils of the leaves, flowers, and stems of the rumberry tree, which can provide antimicrobial, antitumor, and anticholinesterase activities [6].

Rumberries have numerous accessions in germplasm banks with potential use. However, few studies are available related to physical, physicochemical, chemical properties, and fixed compounds present in the fruit. Thus, making it important to research and characterize these aspects, as the lack of information regarding the fruit leads to market devaluation rumberries and its industrialized products. Research that presents new information on the subject can substantiate and encourage its cultivation and consumption in a market with consumers eager for new flavors, natural products, and foods rich in nutritional characteristics such as the rumberry.

This work characterizes the fruit of different accessions of rumberry differentiated by peel color (orange, red, and purple), assessing their physicochemical properties, phenolic compounds, antioxidant activity, and analyzing constituent fixed compounds by paper spray mass spectrometry (PSMS).

## 2. Results and Discussion

### 2.1. Physicochemical Characteristics

Table 1 presents the physical parameters analyzed on fruits of different accessions of rumberry (*Myrciaria floribunda*). Results show the fruits had an average weight of 0.86 g, with a variation of FW (fruit weight) from 0.56 to 1.32 g, for SW (seed weight), the value ranged from 0.13 to 0.30 g, while PW (pulp weight) ranged from 0.42 to 1.12 g.

Earlier studies characterizing rumberry fruits observed lower mean values than those found in this study, reporting mean fruit weights of 0.52, 0.61, and 0.68 g for purple, red and orange colors, respectively [7]. Quality attributes researched in two other genotypes of rumberry trees found higher mean weight values in purple-colored fruits (0.69 g) [3].

Other studies carried out with rumberries show an average reported weight of 0.77 g and 0.14 and 4.0 g for fruits of *Myrciaria tenella* and *Eugenia uniflora* L., respectively [8]. Average weights of around 1.10 g are reported in the literature, a value above that observed in this study [9].

The number of seeds varied from one to two seeds per fruit, accessions AC92, AC112, AC132, AC136, AC137, and AC156 were observed to have two seeds per fruit. Pulp yield (PY) was higher than average for the accessions AC132, AC137, and AC156, at 0.88, 1.12, and 0.89 g, respectively.

Most rumberry fruits have one seed per fruit, with some authors reporting up to two seeds per fruit. However, the number of seeds cannot be relied on as a standard for estimating pulp yields in blackberries, for instance, (*Rubus* spp.), which the number of seeds does not directly correlate with fruit mass [10].

In this study, rumberry fruits with two seeds showed higher pulp yield than fruits with only one seed, except for accession AC92, which was observed to have a 0.47 g yield.

On average, longitudinal diameters (LD) and transverse diameters (TD) were 10.90 and 9.85 mm. Fruits from accessions AC132 and AC156 had the largest LD, reaching 12.76 and 12.00 mm, respectively. For TD, observed values were 11.32 and 11.59 mm. The ratio between the longitudinal and transverse diameter (LD/TD) averaged 1.11 mm, showing that the rumberry fruits have a slightly elliptical or oval shape (LD/TD > 1), with values closer to 1 indicating more roundedness.

Similar results were found in studies evaluating the physical characteristics of rumberry genotypes obtained in the southern region of the State of Alagoas. The variations in the dimensions of LD were observed ranging from 9.30 to 12.18 mm (red and yellow genotypes) and a TD ranging from 8.39 to 10.75 mm (red and purple genotypes). Morphometric characteristics in eleven rumberry accessions native to Alagoas have been reported with an average of 8.86 mm for longitudinal diameter and 9.45 mm for transverse diameter [11]. Lower TD and LD findings with values ranging from 7.51 to 11.90 mm in length and 6.60 to 10.70 mm in width are also observed [8].

Fruit firmness ranged from 1.53 N to 5.93 N, with AC136 standing out for its higher firmness. Aspects of fruit quality of jabuticaba’s trees (*Myrciaria jaboticaba*), a species belonging to the same genus as rumberries, have firmness readings ranging from 3.16 to 5.24 N. This characteristic can be associated with the progressive loss of fruit firmness, as a result in the softening of the pulp. Consequently, the decomposition of cell wall components, breaking down cellulose, hemicellulose, and pectin [12].

Values higher than those found in this study were found when morphologically and chemically characterizing fruits of cambuci (*Campomanesia phaea*), observing a firmness of 19.54 N. Higher firmness of fruits implies fewer losses in quality, thus supporting longer transport chains up until reaching the consumer market. Firmness is, therefore, an important property of fresh fruits [13].

Considering the physical-chemical attributes, rumberry fruit accessions had an average pH of 3.53, as shown in Table 2, considered acidic, i.e., with pH below 4.5. Acidic pH values in rumberries were also observed by other authors who found average pH values of 3.44; 3.90; from 3.87 to 4.37, and from 3.28 to 3.35 [3,7].

Studies carried out with other Myrtaceae also show similar results to those found in this work. Varieties of jabuticaba (*Myrciaria grandifolia* and *Myrciaria jabuticaba*) have been found to have pH values of 3.39 and 3.10, respectively [14]. In cabeludinha (*Myrciaria glazioviana*) fruits, the pH value is reported at around 3.66 [15]. The literature presents data showing myrtle berries (*Eugenia gracillima* Kiaersk.), with a pH of 3.73 and a pH of 3.77 for gabiroba (*Campomanesia adamantium* (Cambess.) O. Berg) [2,16].

Soluble solids content ranged from 11.30 to 14.80 °Brix. These values were similar to findings with 12.96 °Brix for red-colored fruits and 15.76 for yellow-colored fruits [9]. Orange and purple rumberries have been reported in the literature with 12.62 and 16.31 °Brix, respectively [7].

Works with *Eugenia uvalha* Cambess have found values closer to 6.90 °Brix. However, variations in *Campomanesia phaea* fruits are reported ranging from 7.30 to 13.30 °Brix [13]. Physicochemical characteristics of *Myrciaria dubia* fruits from Tarapacá (Amazonas—Colombia) and from the Amazonian fruit tree Germplasm Bank of Agrosavia (Meta—Colombia) have lower values (7.49 and 10.9 °Brix) when compared to those reported in this study, which demonstrates that the contents of soluble solids in Myrtaceae can be quite variable [17].

However, fruits with high levels of °Brix are desirable for consumption in natura and industrialization, as they provide higher yields after processing due to a greater amount of nectar produced per amount of pulp. High soluble solids content also indicates that the fruit has a low preservation potential. The sugars excess may be associated with rapid deterioration and fermentation and, consequently, reduced shelf life. For industrial processing, fruits with higher acidity are used, because it reduces the action of microorganisms and avoids the need for preservatives [18].

Titratable acidity (TA) evaluated in rumberry accessions showed fruits with an average of 4.03% citric acid, with accessions AC132 and AC153 having the highest percentages, with values of 4.78% and 5.65% of citric acid, respectively (Table 2). Results were higher than previously reported values of 0.75% (red), 1.07% (purple) and 1.60% (orange), 1.09% (purple), respectively, and close to that determined by works which reported higher values of 4.97% in fruits of camu-camu (*Myrciaria dubia*) [3,9].

SS/TA ratio showed values ranging from 2.34 (AC153) to 4.55 (AC160), values close to those found in fruits of cambucizeiro (*Campomanesia phaea*) and pitangueira (*Eugenia uniflora* L.) and higher than fruits of camu-camu (*Myrciaria dubia*) [13].

The SS/TA ratio stands out as the best criterion or indicator to evaluate the taste of the fruits. This ratio is more efficient than considering only the levels of sugar and/or acidity quantified in isolation. The balance between sugars with the acids of the fruits gives a taste balance that is determinant for the overall quality of the product.

### 2.2. Total Phenolic Compounds

Phenolic compounds content in different rumberry accessions ranged from 78.79 to 279.01 mg of EAG 100 g^−1^, with AC160 having the highest content of total phenolic compounds, followed by AC137 and AC136 (Table 3). Fruits of the same genus as rumberries have phenolic compound contents of 1480.57 mg of gallic acid L^−1^ as observed in *Myrciaria jabuticaba* and 249.82 mg of gallic acid L^−1^ in *Myrciaria grandifolia* [14].

Camu-camu (*Myrciaria dubia*), another Myrtaceae species, has been found to have values of around 2393.72 mg/100 g of fresh weight. Still, some studies report values ranging from 2625.4 to 1237.86 mg of catechin 100 g^−1^, in fruits of the same species, which can also range down to 369 mg EAG 100 g^−1^ [19].

Levels of phenolic compounds obtained in this study with rumberry were higher than those found in other native fruits from Brazil, such as jabuticaba (128.3 mg EAG 100 g^−1^ of fresh matter), pitanga (95.9 mg EAG 100 g^−1^ of fresh matter), araçá vermelho (88.0 mg EAG 100 g^−1^ of fresh matter), araçá amarelo (72.3 mg EAG 100 g^−1^ of fresh matter), guabiroba (81.7 mg EAG 100g^−1^ of fresh matter), and butia (63.2 mg EAG 100 g^−1^ of fresh matter). Antioxidant activity of extracts of araçá (*Psidium cattleianum*), butia (*Eriospatha butia*), pitanga (*Eugenia uniflora*) and blackberries (*Rubus* sp; cv. Xavante and Cherokee) collected in the southern region of Brazil found total phenolic content ranging from 359.5 to 816.5 mg EAG 100 g^−1^ fresh weight [20]. The content of total phenolic compounds varies according to the type of resource [19].

Fruits are rich in water-soluble compounds, including phenolic compounds. These secondary metabolites are widely used in the food industry to prevent lipid oxidation and reduce cancer incidence [9]. Furthermore, phenolic compounds are associated with a significant contribution to the antioxidant property of fruits [5].

Phenolic compounds take part in flavor perception, shelf life, and biological activity as functional foods, and particularly influence nutritional value and sensory quality, confers color, texture, bitterness, aroma, astringency, and oxidative stability to foods. These compounds are the most abundant antioxidants and are usually found in leaves, seeds, and fruits in varying concentrations. The concentration of these compounds can vary depending on the species, part of the fruit, crop management, geographic and environmental conditions to which they are exposed (sun exposure), and the physiological and genetic factors of the plant.

### 2.3. Chemical Profile by Paper Spray Mass Spectrometry

It was possible to identify 74 compounds in rumberry fruits, comprising flavonoids, phenylpropanoids, ellagitannins, lignans, sugars, fatty acids, phenolic acids, carboxylic acids, benzoic acid derivatives, galotannins, steroids, amines, and cyclitols. Forty-two compounds are identified in the negative ionization mode and 32 in the positive ionization mode, as presented in Table 4 and Table 5. Detected compounds were tentatively identified based on mass fragmentation data compared to those already reported in the literature.

Tentative identification of compounds in the negative ionization mode found flavonoids (16), phenylpropanoids (6), benzoic acid derivatives (7), ellagitannins (3), lignans (3), sugars (2), fatty acid (1), phenolic acid (1), carboxylic acid (1), galotannin (1), and cyclitol (1).

Table 4 shows that among the chemical classes found, flavonoids were the most predominant compounds, especially quercetin-3-glucoside, myricetin rhamnoside, isoquercitrin, hyperin, and myricitrin, which were present in all evaluated accessions.

The ion with [M − H]^−^ at *m*/*z* 463 and its fragmentation of *m*/*z* 301 was tentatively identified as quercetin-3-glucoside, corresponding to the aglycone of quercetin following the loss of a hexose ([M – H − 162]^−^). This result corroborates studies identifying phenolic compounds in grumixama fruit pulp (*Eugenia brasilienses* Lam.) employing HPLC-ESI-MS/MS [21]. This flavonoid was also identified in studies of the chemical profile of grumixama fruits using paper spray mass spectrometry [22].

The ion with [M − H]^−^ at *m*/*z* 463 upon fragmentation led to the formation of aglycone at *m*/*z* 317 ([M – H − 146]^−^) after the loss of one deoxyhexose unit (146 Da), which was tentatively identified as myricetin rhamnoside, rhamnose being the only deoxyhexose found in the fruit flavonoids [23]. Previous studies reported the presence of this substance in jambolão fruit (*Syzygium cumini*) when determining phenolic compounds by HPLC-DAD-MS/MS [24].

**Table 4:** Chemical profile of *Myrciaria floribunda* fruits identified by PS-MS on negative ionization mode.

No	Identification	Formula	*m*/*z* [ ]	MS/MS	Accession									Ref.
					AC67	AC92	AC112	AC132	AC136	AC137	AC153	AC156	AC160	
Flavonoids														
1	Reynoutrin	C_20_H_18_O_11_	433	301	X			X	X	X				[25]
2	quercetin pentoside	-	433	301	X			X	X	X				[26]
3	quercetin 3-*O*-ramnoside (quercitrin)	C_21_H_20_O_11_	447	301	X		X		X				X	[21]
4	myricetin-arabinoside/ xylopyranoside isomer	C_20_H_17_O_12_	449	316, 317		X		X						[25]
5	quercetin-3-glucoside	C_21_H_20_O_12_	463	301	X	X	X	X	X	X	X	X	X	[21]
6	hyperinine	C_21_H_19_O_12_	463	300, 301	X	X	X	X	X	X	X	X	X	[25]
7	myricetin-ramnoside	C_21_H_20_O_12_	463	317	X	X	X	X	X	X	X	X	X	[24]
8	myricitrin	C_21_H_19_O_12_	463	445, 316, 317, 301	X	X	X	X	X	X	X	X	X	[21]
9	quercitrin	C_21_H_20_O_11_	477	301	X		X		X				X	[25]
10	myricetin hexoside isomer	-	479	316, 317	X	X	X		X		X	X	X	[25]
11	Myricetin glucoside	-	479	317	X	X	X		X		X	X	X	[24]
12	Kaempferol-3-*O*-malonylglucoside	-	533	-		X	X	X		X				[27]
13	procyanidin A2	C_30_H_24_O_12_	577	577									X	[28]
14	cyanidin-3-*O*-rutinoside	C_27_H_31_O_15_^+^	593	-				X						[27]
15	methyl-dihydromyricetin diglucoside	-	657	495									X	[24]
16	quercetin derivate	-	867	-	X				X					
Phenylpropanoids														
17	caffeic acid	C_9_H_8_O_4_	179	179, 135	X		X		X	X	X		X	[29]
18	caftaric acid	C_13_H_12_O_9_	311	-	X	X	X	X	X	X	X		X	[27]
19	*p*-coumaric acid hexoside	-	325	183	X		X	X	X	X	X	X	X	[27]
20	caffeoyl-*D*-glucose	-	339	-	X	X		X			X		X	[5,27]
21	caffeoyl hexose	C_15_H_18_O_9_	341	179		X	X			X				[29]
22	chlorogenic acid	C_16_H_18_O_9_	353	353						X				[21,30]
Benzoic acid derivates														
23	ellagic acid glycoside	C_20_H_16_O_13_	721	-									X	[27]
24	ellagic acid pentoside	-	895	-				X						[27]
25	syringic acid hexoside	-	359	197						X				[27]
26	*O*-pentosyl ellagic acid	-	433	301	X			X	X	X				
27	ellagic acid pentoside	-	433	301	X			X	X	X				[25]
28	digaloyl acid	-	339	339	X	X		X			X		X	[30]
29	ellagic acid derivate	-	585	415	X	X			X					[25]
Fatty acids														
30	eicosanoic acid	C_20_H_40_O_2_	311	293	X	X	X	X	X	X	X		X	[29]
Phenolic acids														
31	dimethyl ellagic acid hexoside	C_22_H_22_O_13_	491	475	X			X		X				[27]
Carboxylic acid														
32	citric acid	C_6_H_8_O_7_	191	111	X	X	X	X	X	X	X	X	X	[2,30]
Galotanin														
33	Quercetin galloyl hexoside isomer	-	615	463		X	X	X			X	X		[25]
Ellagitannins														
34	galoyl hexoside ellagic acid	-	615	463, 301		X	X	X			X	X		[21]
35	pedunculagin isomer I	-	783	419				X	X					[31]
36	teligramadine I	C_34_H_26_O_22_	785	301	X	X	X			X				[21]
Lignans														
37	cyclo-lariciresinol Hexoside	-	521	359			X			X				[29]
38	conidendrin	C_20_H_20_O_6_	355	337	X		X	X	X	X	X	X	X	[29]
39	pinoresinol	C_20_H_22_O_6_	357	311	X				X					[31]
Cyclitol														
40	quinic acid	C_7_H_12_O_6_	191	111, 173	X	X	X	X	X	X	X	X	X	[32]
Sugars														
41	sucrose	C_12_H_22_O_11_	377	341			X			X				[27]
42	hexose	C_6_H_12_O_6_	215	179					X					[27]

Compounds identified by other authors in different Myrtaceae species: [28] Cuadrado-Silva, Pozo-Bayón e Osorio. (2017); [24] Faria et al., (2011); [29] Mariano et al., (2020); [31] Mena et al., (2012); [23] Pereira et al., (2017); [27] Rodrigues et al., (2021); [32] Salvador et al., (2011); [25] Santos (2019); [30] Santos et al., (2018); [5] Silva et al., (2019); [21] Teixeira et al., (2015).

**Table 5:** Chemical profile of *Myrciaria floribunda* fruits identified by PSMS on positive ionization mode.

No.	Identification	Formula	*m*/*z* [ ]^+^	MS/MS	Accession									Ref.
					AC67	AC92	AC112	AC132	AC136	AC137	AC153	AC156	AC160	
Flavonoids														
1	catechin	C_15_H_14_O_6_	290	-	X	X		X		X	X	X	X	[33]
2	catechin	C_15_H_14_O_6_	291	273									X	[22]
3	peonidine 3-*O*-glucoside	C_22_H_23_O_11_^+^	301	-				X						[29]
4	diosmetin	C_16_H_12_O_6_	301	-				X						[22]
5	quercetin	C_15_H_10_O_7_	302	-						X				[33]
6	petunidin	C_16_H_13_O_7_^+^(Cl^−^)	317	-	X		X	X	X	X	X	X		[29]
7	miricetin	C_15_H_10_O_8_	318	-		X	X			X	X	X		[33]
8	stigmasterol	C_29_H_48_O	413	-		X								[29]
9	quercetin derivate	-	867	-		X			X					[34]
10	epicatechin gallate	-	442	-									X	[21]
11	cyanidin 3-galactoside	-	449	-		X				X	X		X	[22]
12	myricetin pentoside	-	450	-						X			X	[35]
13	epigallocatechin gallate	C_22_H_18_O_11_	457	-		X		X	X		X			[21]
14	delphinidine hexoside	-	465	303		X								[21]
15	myricetin-ramnoside	-	465	303		X								[27]
16	myricetin-glucoside	-	481	-	X		X	X	X	X		X		[22]
17	myricetin-3-glucoside	-	481	-	X		X	X	X	X		X		[29]
18	myricetin-3-glcA	-	495	-						X				[22]
19	catechin dimer	-	579	-								X		[21]
20	apigenin neohesperidoside I	-	579	-									X	[29]
21	quercetin-3-*O*-[6-(3-hydroxy-3- methyl) glutaroyl-â-galactoside	-	593	-		X		X						[29]
22	rutin	C_27_H_30_O_16_	611	-					X					[33]
23	petunidin-diglucoside		641	-	X	X		X		X	X			[36]
24	cyanidin-3-(p-hydroxybenzoyl)- (oxaloyl)diglucoside-5-glucoside		970	-				X						[35]
Benzoic acid derivates														
25	Galloylpyrogallol	-	279	-									X	[29]
26	galloyl-glucose esther	-	355	-									X	[22]
27	ellagic acid hexoside	-	927	-	X			X						[21]
Sugars														
28	glucose	C_6_H_12_O_6_	219	-	X	X	X	X	X	X	X	X	X	[27]
29	sucrose/hexose	-	381	219	X	X	X	X		X	X	X	X	[27]
Steroids														
30	stigmasterol	C_29_H_48_O	413	-		X								[35]
Phenylpropanoids														
31	dihydrosynapic acid	-	475	457		X								[22]
Amines														
32	gomfrenin	-	551	-		X			X	X	X			[37]

Compounds identified by authors in different Myrtaceae species: [34] Alves et al., (2017); [37] García-Cruz et al., (2021); [36] Li, Zhang e Seeram. (2009); [29] Mariano et al., (2020); [22] Ramos et al., (2020); [27] Rodrigues et al., (2021); [35] Sharma et al., (2015); [33] Siebert et al., (2017); [21] Teixeira et al., (2015).

While in the composition of myrtle (*Myrtus communis* L.) leaf extracts obtained through different extraction techniques (supercritical extraction, conventional extraction, and ultrasonic extraction) employing HPLC-MS, differences were observed in the types of compounds obtained. The compound myricetin rhamnoside was nevertheless present in all extracts regardless of the technique used.

Studies of plant species native to Brazil from four different genera (*Plinia*, *Myrciaria*, *Psidium*, and *Eugenia*) of Myrtaceae, identified the ion at *m*/*z* 463 as hyperin for the species *Myrciaria floribunda* and *Myrciaria glomerata*. In contrast, for the species *Eugenia uniflora* the compound was identified as isoquercitrin, and both ions showed identical mass fragmentation (*m*/*z* 301 and 300) [25].

The peak *m*/*z* 447 signal points to the presence of a rhamnoside unit, suggesting identifying the substance as quercetin 3-*O*-ramnnoside (quercitrin) upon fragmentation at *m*/*z* 301. The occurrence of this flavonoid (quercitrin) has been previously recorded in Myrtaceae species, as reported in fruits of the species *Eugenia invlucrata* DC and fruits of the species *Eugenia punicifolia* [22,26].

The antioxidant capacity of leaves found in *Eugenia chlorophylla*, *Eugenia pyriformis*, *Myrcia laruotteana*, and *Myrcia obtecta* species reported two of the flavonoids identified in this work (myricitrin and quercitrin) with antioxidant and antimicrobial activities. Furthermore, associated quercetin with antiglycant activity [32].

Flavonoids are phenolic compounds synthesized by plants as secondary metabolites. These metabolites are found in plants’ leaves, flowers, roots, and fruits in glycosylated form and may or may not be attached to sugars (rhamnose, rutinose, and glucose). Flavonoids act in plants as protection against UV radiation, protection against microorganisms, enzyme inhibition, antioxidant and antibiotic action, and reproductive functions because the colored compounds, such as the red and blue colors present in some flowers, attract pollinating insects [38].

Phenolic compounds have become of great interest in research due to their beneficial health properties, among which is a great antioxidant capacity to delay or inhibit the oxidation of molecules inside the human body that cause stress and some diseases, such as cancer. Furthermore, phenolic compounds possess biological activities with effects such as antimicrobial, anti-inflammatory, and vasodilatory action within the body [38,39].

Phenylpropanoids which were most abundant in the rumberry fruits were tentatively identified as caffeic acid (*m*/*z* 179), caftaric acid (*m*/*z* 311), *p*-coumaric acid hexoside (*m*/*z* 325), caffeoyl-D glucose (*m*/*z* 339), caffeoyl hexose (*m*/*z* 341), and chlorogenic acid (*m*/*z* 353), the latter only found in fruits of accession AC137. Orange fruits had the highest number of compounds detected.

The peak with *m*/*z* 179 had a fragmentation pattern of *m*/*z* 179 and *m*/*z* 135 [M − H-44]^−^ identified as caffeic acid, resulting from the loss of a mass of 44 u, corresponding to one carbonyl group unit. The [M − H]^−^ ion of *m*/*z* 311 was identified as caftaric acid, corroborating the studies performed a study where changes in bioactive compounds from cagaita pulp were evaluated (*Eugenia dysenterica*) [27].

The [M − H]^−^ ion of *m*/*z* 325 showed MS/MS fragmentation at *m*/*z* 183, suggesting the identification of the substance as a *p*-coumaric acid hexoside, which corresponds to a hydroxycinnamic acid conjugated to a hexose [5]. The [M − H]^−^ ion of *m*/*z* 353 showed *m*/*z* of 353 as the MS/MS fragmentation pattern, thus proposing chlorogenic acid as the signature compound.

Phenolic compounds identified as caffeic acid and chlorogenic acid showed considerable antioxidant activity in the extracts analyzed from *Myrciaria* and *Eugenia* plants [32]. In addition, other studies have reported antimutagenic, anticancer, and anti-inflammatory properties [40].

The peak at *m*/*z* 339 was classified as caffeoyl-*D*-glucose due to the conjugated form of caffeic acid with a hexose. This conjugated substance in cagaita fruits (*Eugenia dysenterica*) was also found employing paper spray mass spectrometry (PSMS) [41].

Compounds derived from benzoic acids were the third most predominant class in the composition of the chemical profile of the rumberry fruit. Compounds were identified as an ellagic acid glycoside, ellagic acid pentoside, syringic acid hexoside, *O*-pentosyl ellagic acid, ellagic acid pentoside, digaloyl acid, and ellagic acid derivative, which presented the ions with peaks at *m*/*z* 721, 895, 359, 433, 339, and 585.

The [M − H]^−^ ion of *m*/*z* 433 presented the fragmented ion at *m*/*z* 301, thus being identified as ellagic acid pentoside. The identification of this substance was confirmed by works carried out in *Plinia edulis*, *Eugenia luschnatiana,* and *Syzygium jambos*. This compound is also identified at 895 *m*/*z* [27].

Three classes of acids were identified in the rumberry fruit, corresponding to the compounds oxo-dihydroxy eicosanoic acid (fatty acid), dimethyl ellagic acid hexoside (phenolic acid), and citric acid (carboxylic acid). The peak with *m*/*z* 311 and fragment *m*/*z* 293 was identified as eicosanoic acid, as with findings on pera do cerrado constituents (*Eugenia klotzschiana*) [29].

The peak at *m*/*z* 491 has not yet been reported in rumberry fruits. However, further studies identified this ion as a hexoside of dimethyl ellagic acid for *Eugenia dysenterica* [27]. This substance contributes to the characterization of flavor. Some studies have associated phenolic acids such as this compound with high antioxidant capacity, antimutagenic properties, anticarcinogenic, and the ability to alter gene expression.

The ion with [M − H]^−^ at *m*/*z* 191 can be identified as citric acid, a carboxylic acid that was tentatively identified based on its MS/MS fragmentation pattern at *m*/*z* 111 [M − H-80]^−^, being consistent with data described in the scientific literature [5,38,40].

The three ellagitannins are prominent in rumberries corresponded to the pedunculagin I isomer, telimagrandin I, and ellagic acid galoyl hexoside. Identification of ions was confirmed by MS/MS fragmentation pattern. The *m*/*z* 615 ion upon MS/MS fragmentation at *m*/*z* 463 and *m*/*z* 301 was tentatively identified as galoyl hexoside ellagic acid, resulting from the loss of a galoyl group and a hexose unit (162). Whereas the [M − H]^−^ ion of *m*/*z* 785 was identified as telimagrandin I by its MS/MS fragmentation at *m*/*z* 301, characteristic of the loss of the galoyl group from casuarictin. As for the [M − H]^−^ ion at *m*/*z* 783, was identified by its MS/MS fragment at *m*/*z* 419 as a pedunculagin I isomer.

The presence of these compounds in grumixama fruits (Myrtaceae: *Eugenia brasiliensis* Lam.), employing HPLC-ESI-MS/MS. Myrtaceae is known to have species-rich in ellagitannins (ETs), which have demonstrated antioxidant activities and antimutagenic and anticarcinogenic effects [21].

Substances classified as lignans correspond to the [M − H]^−^ ions of *m*/*z* 521, *m*/*z* 355, and *m*/*z* 357 tentatively identified as lariciresinol, conidendrin, and pinoresinol cycle hexoside, respectively. These compounds have been previously described in species of *Eugenia klotzschiana* and *Punica granatum* L. [29].

The [M − H]^−^ ion of *m*/*z* 191 suggests the presence of quinic acid, or scopoletin acid, or citric acid. However, ion identification was only achieved after its fragmentation at *m*/*z* 111 and *m*/*z* 173. In the case of scopoletin, the substance produced a single fragmented ion at *m*/*z* 176 and was thus disregarded as there was no evidence of this fragment. On the other hand, citric acid showed the same fragment at *m*/*z* 111 as quinic acid and was, therefore, differentiated by its fragmented ion at *m*/*z* 173, which gives it the name quinic acid. This substance has been confirmed previously by other authors [38,40].

According to the fragmentation profile presented in Table 4, the ions with *m*/*z* 215 and *m*/*z* 377 were identified by their fragments at *m*/*z* 179 and *m*/*z* 341, respectively, as hexose and sucrose, compounds recognized as sugars. Sucrose was also identified in cagaita pulp with peel, with reports of these two sugars for the same species [27].

As for the tentative identification of compounds in the positive ionization mode, the chemical profile of *Myrciaria floribunda* fruits was represented by flavonoids (24), benzoic acid derivatives (3), sugars (2), steroids (1), phenylpropanoids (1), and amines (1).

Among the chemical classes found, flavonoids were the most abundant as with negative ionization mode, with catechin (*m*/*z* 290), petunidin (*m*/*z* 317), myricetin (*m*/*z* 318), myricetin-glucoside (*m*/*z* 481), myricetin-3-glucoside (*m*/*z* 481), and petunidin-diglucoside (*m*/*z* 641) as the compounds that stood out the most as they were identified in most of the accessions (Table 5).

Catechin and cyanidin 3-galactoside were the only compounds that were present in the orange (AC67, AC92, AC137, and AC156), red (AC132 and AC153), and purple (AC160) fruit varieties. Catechin has been described previously in grumixama leaves (*Eugenia brasiliensis*), identified by the [M − H]^+^ ion of *m*/*z* 290. Pulps of the same species had the presence of catechin identified with the ion at *m*/*z* 291 and fragment at *m*/*z* 273, another species of the Myrtaceae family [22,33].

The presence of catechins in the extracts of red and yellow araçá trees have been found with 36,225 and 34,396, 20,748 mg g^−1^, respectively [42]. For the jabuticabeira hybrid, catechin quantification was lower (20,748 mg g^−1^). Studies have reported the catechin as possessing therapeutic effects due to its health-related benefits, such as antioxidant, antifungal, antitumor properties as well as an insect repellent [43]. Catechins are one of the most studied antioxidants [44].

Detection of the cyanidin 3-galactoside identified by the ion with *m*/*z* 449 confirms the presence of anthocyanins in rumberry fruits [22]. This anthocyanin is classified as a flavonoid and has been reported previously in camu-camu fruits (*Myrciaria dubia*), with values of 30–54 mg/100 g fresh peel and in jabuticaba fruits (*Myrciaria cauliflora*), with values of 1.6 to 2.1 g/100 g dry weight [45].

As for benzoic acid derivates, purple fruits (AC160) showed the compounds galoylpyrogallol *(m*/*z* = 279) and galoyl-glucose esther (*m*/*z* = 355), while orange (AC67) and red (AC132) fruits had the compound ellagic acid hexosides (*m*/*z* = 927). These compounds have already been reported in fruits of other Myrtaceae, such as the species *Eugenia klotzschiana* and *Eugenia brasiliensis* [21,22,29].

Ions with [M − H]^+^ at *m*/*z* 219 and *m*/*z* 381 were tentatively identified as sugars. Sucrose was detected with the highest concentration in *Campomanesia lineatifolia* fruits, followed by fructose and glucose [46].

A steroid and a phenylpropanoid were also identified only in orange-colored fruits (AC67). The steroid with [M − H]^+^ at *m*/*z* 413 corresponded to the compound identified as stigmasterol, which was reported in studies with jamun fruits (*Eugenia jambolana*) [35]. The ion with [M − H]^+^ at *m*/*z* 475 corresponded to the compound dihydrosynaptic acid, identified in other works with Myrtaceae by its fragment at *m*/*z* 457 [22].

Some phenylpropanoids, such as eugenol, have demonstrated various pharmacological activities, such as anti-inflammatory, antitumor, antibacterial, antifungal, antipyretic, anesthetic, and analgesic activities [47]. Studies have also revealed that many chronic diseases such as cardiovascular diseases, type II diabetes, and various types of cancer can have their chance of development reduced with the use of phenylpropanoids [48].

## 3. Materials and Methods

### 3.1. Sample Acquisition

Ripe fruits of rumberry (*Myrciaria floribunda* (H. West ex Willd.) O. Berg) of different accessions, differentiated by their orange (AC67, AC92, AC112, AC136, AC137, AC156), red (AC132, AC153), and purple (AC160) coloration were collected from the Active Germplasm Bank of Cambuí (BAG—Cambuí, Brazil) of the Federal University of Alagoas (UFAL), Rio Largo, State of Alagoas (latitude 09°28′42″ S, longitude 35°51′12″ W, altitude 127 m). According to the Köppen classification, the climate of the region is type “As”, i.e., tropical climate with the rainy season in the fall/winter and dry in the summer, average annual precipitation of 1150.2 mm, with November and December being the driest months and July and August the rainiest [49].

Fully ripened fruits were hand-harvested in the early morning hours. After harvesting, they were stored in polyethylene bags, labeled, and packed in insulated boxes with ice for later transport to the Plant Biotechnology Laboratory of CECA/UFAL. In order to eliminate impurities, where they were selected according to the physiological maturation stage, washed in running water, and sanitized with 20 mL of sodium hypochlorite (1%).

### 3.2. Physicochemical Characterization

For physical analyses, 128 fruits from each of the nine accessions were evaluated. Fruit weight (FW), seed weight (SW), and pulp weight (PW) were evaluated in grams; longitudinal diameter (LD) and transverse diameter of the fruit (TD) were expressed in millimeters, determined using an analytical balance (BEL Engineering^®^-Mark 1300—Monza, Milano, Italy) with the precision of 0.01 g and digital pachymeter (Jomarca-150 mm) with a sensitivity of 0.01 mm; Number of seeds per fruit (NS) was determined by manual counting, and fruit firmness was determined with a digital penetrometer (Instrutherm PTR-300) with a 3 mm diameter tip, with results expressed in Newton (N).

After measuring, fruits were stored in the freezer at −18 °C until the physical-chemical analyses. In order to determine pH, SS, AT, and SS/AT ratio, *M. floribunda* fruits were taken from the freezer and left to thaw at room temperature. The pulp was then extracted with a mortar, and seeds were discarded.

A digital pH meter (model Mfa-210) was used, calibrated with pH 4.0 and 7.0 buffer solutions, according to the Adolfo Lutz Institute methodology. Soluble solids (SS) were quantified in a digital HAND-HELD pocket refractometer (model PDR-50B), with results expressed in °Brix, according to AOAC method 932.12. Titratable acidity (TA) was quantified by titration, transferring to a 250 mL Erlenmeyer flask approximately 2.0 g of pulp, which was diluted in 50 mL of distilled water and added three drops of phenolphthalein and then titrated with 0.1 N NaOH solution. Results were expressed in g of citric acid/100 mL (%). The SS/AT ratio was determined by the quotient between the values of soluble solids and titratable acidity.

### 3.3. Total Phenolic Compounds and Paper Spray Mass Spectrometry

Samples of 0.5 g of previously homogenized rumberry pulp were added in 2 mL Eppendorf wrapped with aluminum foil, to which 1 mL of methanol/water solution (50:50, *v*/*v*) was added. The sample was mixed on a vortex for 20 s and incubated for 1 h at room temperature, protected from light. After the incubation time, samples were centrifuged for 15 min at 4 °C with a rotation of 15,000 rpm, and the obtained supernatant was transferred to the same 5 mL volumetric flask. Subsequently, 1 mL of acetone/water (70:30, *v*/*v*) was added to the same Eppendorf vial, and the complete homogenization and resting process was repeated. Solutions were centrifuged again under the conditions mentioned earlier, and the supernatant obtained was then transferred again to the volumetric flask (5 mL) of the first supernatant. They were then mixed, and the volume was completed with deionized water [50].

Extracts were stored in 2 mL Eppendorf vials at freezing temperature until the time of use to determine the phenolic compound content and PS-MS chemical profiling.

#### 3.3.1. Total Phenolics

A volume of 300 µL of sample extracts, 3.5 mL of distilled water, and 250 µL of Folin-Ciocalteu reagent were mixed in a test tube and incubated at room temperature for 3 min, then 500 µL of sodium carbonate solution (7.5%) was added. After 1 h of incubation in the dark, the samples were read at 750 nm, and the data were expressed in mg gallic acid (EAG)/100 g^−1^ of pulp. Total phenolic compounds were quantified in a spectrophotometer at 750 nm absorbance, using a standard curve prepared with gallic acid (GAE—gallic acid equivalent).

#### 3.3.2. Paper Spray Mass Spectrometry

Pulps were analyzed using an ambient paper spray ionization source in a Thermo Fisher LCQ FLEET ion-trap mass spectrometer (Thermo Scientific, San Jose, CA, USA). Analyses were performed in triplicate in positive and negative ionization modes. For PS-MS analyses, a 2 µL sample volume and 40 µL of methanol were added to a triangular chromatographic paper (equilateral, 1.5 cm lengthwise) positioned 10 mm away from the mass spectrometer inlet [5,22,51].

A high voltage was then applied to the paper held by a copper clamp and attached to a three-dimensional moving platform for data acquisition. Instrumental conditions of operation were: PSMS source voltage equal to +4 kV (positive mode) and −3 kV (negative mode); capillary voltage of 40 V; transfer tube temperature of 275 °C; tube lens voltage of 120 V; mass scanning range of 100 to 1000 *m*/*z* in positive and negative modes.

Ions were fragmented using collision energies from 15 to 45 eV. Tentative identification of the compounds was carried out using a comparison of the *m*/*z* ratios of the data obtained from the literature associated with instrumental readings and subsequent fragmentation employing sequential mass spectrometry.

### 3.4. Statistics

The experimental design was entirely randomized for physical and physical-chemical analyses, each treatment consisted of four repetitions of thirty-two fruits for each accession. Results were submitted to analysis of variance using the software Sisvar version 5.7. Means were compared using the Scott–Knott test (*p* ≤ 0.05). Total phenolic compounds were evaluated using central tendency measurement, using Microsoft Office Excel^®^ v. 16.0 (Microsoft, Redmond, WA, USA). PS-MS spectra, both positive and negative ionization mode, were processed using Xcalibur v. 2.1 (Thermo Scientific, San Jose, CA, USA) and Microsoft Office Excel^®^.

## 4. Conclusions

Rumberry fruits of the different accessions evaluated are relatively small and oval-shaped, each containing 1 to 2 seeds. Accessions AC132, AC137, and AC156 had higher fruit weight, and accessions AC132 and AC156 were largest by size. Access AC137 had the highest fruit pulp yield. Fruits from access AC160 had a higher soluble solids/titratable acidity ratio and higher total phenolic content.

The chemical profile of rumberry fruits using paper spray mass spectrometry proved to be fast and efficient since several phenolic compounds responsible for the antioxidant and phenolic activity of the fruits were identified. However, orange-colored fruits stood out among other accessions, as in both negative and positive ionization, flavonoids were the main identified constituent compounds.

Physical, physicochemical, and chemical composition was similar to that of fruits from other species of the Myrtaceae family. Such characteristics serve as a reference in future works of characterization of rumberry. Further studies should be carried out to improve knowledge of its agro-industrial characteristics.

## Figures and Tables

**Table 1:** Physical characteristics of fruits of nine accessions of *Myrciaria floribunda*, differentiated by their orange, red, and purple coloration.

Accessions	Parameters							
	FW (g)	SW (g)	PW (g)	NS	LD (mm)	TD (mm)	Shape (mm)	Firmness (N)
AC-67	0.73 ^a^	0.18 ^b^	0.55 ^a^	1.37 ^a^	10.71 ^b^	9.75 ^b^	1.10 ^c^	1.53 ^a^
AC-92	0.61 ^a^	0.14 ^a^	0.47 ^a^	1.52 ^b^	9.86 ^a^	8.56 ^a^	1.15 ^e^	2.36 ^b^
AC-112	0.83 ^a^	0.24 ^c^	0.58 ^a^	1.73 ^b^	10.96 ^b^	10.02 ^b^	1.09 ^c^	4.16 ^c^
AC-132	1.18 ^b^	0.30 ^d^	0.88 ^a^	1.67 ^b^	12.76 ^d^	11.32 ^c^	1.13 ^d^	1.75 ^a^
AC-136	0.76 ^a^	0.18 ^b^	0.59 ^a^	1.52 ^b^	11.03 ^b^	9.84 ^b^	1.12 ^d^	5.93 ^d^
AC-137	1.32 ^b^	0.20 ^b^	1.12 ^a^	1.71 ^b^	11.06 ^b^	9.61 ^b^	1.15 ^e^	3.56 ^c^
AC-153	0.56 ^a^	0.14 ^a^	0.42 ^a^	1.15 ^a^	9.75 ^a^	9.12 ^a^	1.07 ^b^	4.50 ^c^
AC-156	1.18 ^b^	0.29 ^c^	0.89 ^a^	1.78 ^b^	12.00 ^c^	11.59 ^c^	1.04 ^a^	2.80 ^b^
AC-160	0.57 ^a^	0.13 ^a^	0.45 ^a^	1.01 ^a^	9.94 ^a^	8.85 ^a^	1.12 ^c^	4.17 ^c^
Mean	0.86	0.20	0.66	1.49	10.90	9.85	1.11	3.42
CV (%)	43.62	17.08	57.43	14.12	4.08	4.50	1.04	16.80
Standard Error	0.19	0.02	0.19	0.11	0.22	0.22	0.01	0.29

Scott–Knott test. Means followed by the same letter in the column do not differ statistically (*p* ≤ 0.05). FW: fruit weight; SW: seed weight; PW: pulp weight; NS: number of seeds; LD: longitudinal diameter; TD: transversal diameter; shape: relation between the variables LD/TD.

**Table 2:** Physicochemical characteristics of fruits from nine accessions of *Myrciaria floribunda*, differentiated by their orange, red, and purple coloration.

Accessions	pH	SS	AT	SS/AT
AC-67	3.40 ^a^	14.10 ^c^	3.80 ^b^	3.84 ^c^
AC-92	3.58 ^b^	13.13 ^b^	4.23 ^b^	3.11 ^b^
AC-112	3.70 ^b^	12.90 ^b^	4.13 ^b^	3.13 ^b^
AC-132	3.26 ^a^	11.30 ^a^	4.78 ^c^	2.38 ^a^
AC-136	3.43 ^a^	14.80 ^c^	3.35 ^a^	4.45 ^d^
AC-137	3.61 ^b^	14.80 ^c^	3.78 ^b^	3.95 ^c^
AC-153	3.48 ^a^	13.18 ^b^	5.65 ^d^	2.34 ^a^
AC-156	3.61 ^b^	12.85 ^b^	3.48 ^a^	3.71 ^c^
AC-160	3.70 ^b^	13.70 ^c^	3.05 ^a^	4.55 ^d^
Mean	3.53	13.42	4.03	3.49
CV (%)	3.27	7.30	9.01	14.48
Standard Error	0.06	0.49	0.18	0.25

Scott–Knott test. Means followed by the same letter in the column can be grouped together (*p* ≤ 0.05). pH: potential of hydrogen; SS: soluble solids (°Brix); TA: titratable acidity (% of citric acid); SS/AT: ratio between the two variables.

**Table 3:** Mean values and standard deviation of values of total phenolic compounds in different rumberry accessions.

Accessions	Total Phenolics (mg Gallic Acid. 100 g^−1^ of Fresh Matter)
AC-67	162.33 ± 1.52
AC-92	136.79 ± 4.97
AC-112	78.79 ± 1.52
AC-132	202.85 ± 6.35
AC-136	204.77 ± 0.88
AC-137	254.70 ± 8.19
AC-153	128.91 ± 2.30
AC-156	115.95 ± 8.35
AC-160	279.01 ± 11.11

Data are presented as the means of the results of triplicate sample analysis ± standard deviation.

## Data Availability

All data is contained within the article.

## References

[1] García Y.M., Ramos A.L.C.C., De Paula A.C.C.F.F., Do Nascimento M.H., Augusti R., De Araújo R.L.B., De Lemos E.E.P., Melo J.O.F. (2021). Chemical Physical Characterization and Profile of Fruit Volatile Compounds from Different Accesses of *Myrciaria floribunda* (H. West Ex Wild.) O. Berg through Polyacrylate Fiber. Molecules.

[2] De Araujo D.R., De Lucena E.M.P., Gomes J.P., de Figueirêdo R.M.F., Silva E. (2015). Características físicas, químicas e físico-químicas dos frutos da murta. Rev. Verde Agroecol. E Desenvolv. Sustentável.

[3] Silva A.V.C., Nascimento A.L.S., Muniz E.N. (2020). Fruiting and quality attributes of cambui (*Myrciaria floribunda* (West ex Willd.) O. Berg in the Atlantic Forest of northeast Brazil. Rev. Agro@Mbiente Line.

[4] García Y.M., Rufini J., Campos M.P., Guedes M.N., Augusti R., Melo J.O. (2019). SPME fiber evaluation for volatile organic compounds extraction from acerola. J. Braz. Chem. Soc..

[5] Silva M.R., Freitas L.G., Souza A.G., Araújo R.L., Lacerda I.C., Pereira H.V., Augusti R., Melo J.O. (2019). Antioxidant activity and metabolomic analysis of cagaitas (*Eugenia dysenterica*) using paper spray mass spectrometry. J. Braz. Chem. Soc..

[6] Tietbohl L.A., Barbosa T., Fernandes C.P., Santos M.G., Machado F.P., Santos K.T., Rocha L. (2014). Laboratory evaluation of the effects of essential oil of *Myrciaria floribunda* leaves on the development of Dysdercus peruvianus and Oncopeltus fasciatus. Rev. Bras. De Farmacogn..

[7] Rezende L., Almeida C.S., Da Silva A.V. (2011). Diversidade genética de uma população natural de cambuizeiro e avaliação pós-colheita de seus frutos. Sci. Plena.

[8] Da Silva A.V.C., Rabbani A.R.C., Costa T.S., Clivati D. (2012). Fruit and seed biometry of cambuí (*Myciaria tenella* O. Berg). Rev. Agro@ Mbiente Line.

[9] Dos Santos E.F., De Lemos E.E.P., De Lima Sanvador T., De Araújo R.R. (2017). Caracterização físico-química, compostos bioativos e atividade antioxidante total de frutos de cambuizeiro (*Myrciaria floribunda* O. Berg). Rev. Ouricuri.

[10] Figueiredo M.A.D., Pio R., Silva T.C., Silva K.N. (2013). Características florais e carpométricas e germinação In Vitro de grãos de pólen de cultivares de amoreira-preta. Pesqui. Agropecuária Bras..

[11] Araújo R.R.D. (2012). Qualidade e potencial de utilização de frutos de genótipos de Cambuí, Guajiru e Maçaranduba nativos da vegetação litorânea de Alagoas. 174 f. Tese. Ph.D. Thesis.

[12] Semensato L.R., Vendruscolo E.P., Seleguini A., Batista Filho P.A., da Silva E.C.M., da Silva T.P. (2020). Fenologia, produtividade e qualidade de frutos de jabuticabeiras de diferentes idades das plantas. Iheringia. Série Botânica..

[13] Bianchini F.G., Balbi R.V., Pio R., Silva D.F.D., Pasqual M., Vilas Boas E.V.D.B. (2015). Caracterização morfológica e química de frutos de cambucizeiro. Bragantia.

[14] Almeida E.S., Silva R.J.N., Gonçalves E.M. (2018). Compostos fenólicos totais e características físico-químicas de frutos de jabuticaba. Gaia Sci..

[15] De Mendonga V.Z., Vieites R.L. (2019). Physical-chemical properties of exotic and native Brazilian fruits. Acta Agronómica.

[16] Da Conceição Souza J.L., Borges L., Reges N.P.R., Mota E.E.S., Leonídio R.L. (2019). Caracterização física e química de gabiroba e murici. Rev. Ciências Agrárias.

[17] Aguirre-Neira J.C., Reis M.S.D., Cardozo M.A.R., Raz L., Clement C.R. (2020). Physical and chemical variability of Camu-camu fruits in cultivated and uncultivated areas of the Colombian Amazon. Rev. Bras. De Frutic..

[18] Lattuada D.S., Pezzi E., de Souza P.V.D. (2018). Caracterização de frutos em diferentes estádios de maturação de um Guapuritizeiro. Pesqui. Agropecuária Gaúcha.

[19] Jáuregui A.M.M., Ramos-Escudero D.F., Ureta C.A.-O., Castañeda B.C. (2007). Evaluación de la capacidad antioxidante y contenido de compuestos fenólicos en recursos vegetales promisorios. Rev. Soc. Química Perú.

[20] De Souza A.G., Fassina A.C., Saraiva F.R.d.S., De Souza L. (2018). Caracterização físico-química de frutos nativos da região Sul do Brasil. Evidência.

[21] Teixeira L.D.L., Bertoldi F.C., Lajolo F.M., Hassimotto N.M.A. (2015). Identification of ellagitannins and flavonoids from *Eugenia brasilienses* Lam.(Grumixama) by HPLC-ESI-MS/MS. J. Agric. Food Chem..

[22] Ramos A.L.C.C., Mendes D.D., Silva M.R., Augusti R., Melo J.O.F., de Araújo R.L.B., Lacerda I.C.A. (2020). Chemical profile of *Eugenia brasiliensis* (Grumixama) pulp by PS/MS paper spray and SPME-GC/MS solid-phase microextraction. Res. Soc. Dev..

[23] Pereira P., Cebola M.J., Oliveira M.C., Gil M.G.B. (2017). Antioxidant capacity and identification of bioactive compounds of *Myrtus communis* L. extract obtained by ultrasound-assisted extraction. J. Food Sci. Technol..

[24] Faria A.F., Marques M.C., Mercadante A.Z. (2011). Identification of bioactive compounds from jambolão (*Syzygium cumini*) and antioxidant capacity evaluation in different pH conditions. Food Chem..

[25] Santos L.D.S. (2019). Compilação de dados de composição nutricional e quimiotaxonomia de espécies da família myrtaceae por UPLC-MS acoplada à quimiometria. Tese de Doutorado.

[26] Nicacio A.E., Rotta E.M., Boeing J.S., Barizao E.O., Kimura E., Visentainer J.V., Maldaner L. (2017). Antioxidant activity and determination of phenolic compounds from *Eugenia involucrata* DC. Fruits by UHPLC-MS/MS. Food Anal. Methods.

[27] Rodrigues D., Mendonça H., Nogueira L. (2021). Caracterização de compostos voláteis e compostos bioativos da polpa e geleia de cagaita por microextração em fase sólida no modo headspace e espectrometria de massa por paper spray. Res. Soc. Dev..

[28] Cuadrado-Silva C.T., Pozo-Bayón M.Á., Osorio C. (2017). Targeted metabolomic analysis of polyphenols with antioxidant activity in sour guava (*Psidium friedrichsthalianum* Nied.) fruit. Molecules.

[29] Mariano A.P.X., Ramos A.L.C.C., Augusti R., Araújo R.L.B., Melo J.O.F. (2020). Analysis of the chemical profile of cerrado pear fixed compounds by mass spectrometry with paper spray and volatile ionization by SPME-HS CG-MS. Res. Soc. Dev..

[30] Santos E.F.D. (2018). Caracterização fenológica e morfológica de plantas e qualidade pós-colheita de frutos de acessos de cambuizeiro (*Myrciaria floribunda* O. Berg) do banco ativo de germoplasma do CECA-UFAL. Dissertação de Mestrado.

[31] Mena P., Calani L., Dall’Asta C., Galaverna G., García-Viguera C., Bruni R., Crozier A., Del Rio D. (2012). Rapid and comprehensive evaluation of (poly) phenolic compounds in pomegranate (*Punica granatum* L.) juice by UHPLC-MSn. Molecules.

[32] Salvador M.J., De Lourenço C.C., Andreazza N.L., Pascoal A.C., Stefanello M.É.A. (2011). Antioxidant capacity and phenolic content of four Myrtaceae plants of the south of Brazil. Nat. Prod. Commun..

[33] Siebert D.A., Bastos J., Spudeit D.A., Micke G.A., Alberton M.D. (2017). Determination of phenolic profile by HPLC-ESI-MS/MS and anti-inflammatory activity of crude hydroalcoholic extract and ethyl acetate fraction from leaves of *Eugenia brasiliensis*. Rev. Bras. Farmacogn..

[34] Alves A.M., Dias T., Hassimotto N.M.A., Naves M.M.V. (2017). Ascorbic acid and phenolic contents, antioxidant capacity and flavonoids composition of Brazilian Savannah native fruits. Food Sci. Technol..

[35] Sharma R.J., Gupta R.C., Bansal A.K., Singh I.P. (2015). Metabolite Fingerprinting of Eugenia jambolana Fruit Pulp Extracts using NMR, HPLC-PDA-MS, GC-MS, MALDI-TOF-MS and ESI-MS/MS Spectrometry. Nat. Prod. Commun..

[36] Li L., Zhang Y., Seeram N.P. (2009). Structure of anthocyanins from *Eugenia jambolana* fruit. Nat. Prod. Commun..

[37] García-Cruz L., Guerra-Ramírez D., Martínez-Damián M.T., Zuleta-Prada H., Valle-Guadarrama S. (2021). Shelf Life of Pitaya (*Stenocereus pruinosus*) Fruits Affected by Temperature and The Use Of Biopolymers Of Guar Gum. Acta Agrícola Y Pecu..

[38] de Oliveira Júnior A.H., Ramos A.L.C.C., Guedes M.N.S., Fagundes M.C.P., Augusti R., Melo J.O.F. (2020). Chemical profile and bioprospecting of cocoa beans analyzed by paper spray mass spectrometry. Res. Soc. Dev..

[39] Do Nascimento C.D., De Paula A.C., De Oliveira A.H., Mendonça H.D.O., Reina L.D.C., Augusti R., Figueiredo-Ribeiro R.d.C.L., Melo J.O. (2021). Paper Spray Mass Spectrometry on the Analysis of Phenolic Compounds in *Rhynchelytrum repens*: A Tropical Grass with Hypoglycemic Activity. Plants.

[40] García Y.M., de Lemos E.E.P., Augusti R., Melo J.O.F. (2021). Optimization of extraction and identification of volatile compounds from *Myrciaria floribunda*. Rev. Ciência Agronômica.

[41] Silva M.R., De Souza A.G., De Araújo R.L.B., Lacerda I.C.A., Augusti R., Melo J.O.F., Mendonça H.D.O.P. (2020). Análise metabolômica de cagaitas utilizando a espectrometria de massas com ionização por paper spray. Avanços Ciência E Tecnol. Alimentos. Científica.

[42] Branco L.A., de Oliveira H.L.M., de Campos Bortolucci W., Fernandez C.M.M., Gonçalves J.E., Gazim Z.C., Junior R.P. (2020). Control of bovine tick (*Rhipicephalus microplus*) with essential oil from Psidium rufum DC leaves. Res. Soc. Dev..

[43] Guedes M.N.S., Pio R., Maro L.A.C., Lage F.F., Abreu C.M.P.D., Saczk A.A. (2017). Antioxidant activity and total phenol content of blackberries cultivated in a highland tropical climate. Acta Scientiarum. Agron..

[44] Grzesik M., Naparło K., Bartosz G., Sadowska-Bartosz I. (2018). Antioxidant properties of catechins: Comparison with other antioxidants. Food Chem..

[45] Zhang J., Celli G.B., Brooks M.S. (2019). Natural sources of anthocyanins. R. Soc. Chem..

[46] Balaguera-López H.E., Herrera Arevalo A. (2012). Biochemical changes during growth and until harvest of champa (*Campomanesia lineatifolia* R. & P. Myrtaceae family) fruit. Rev. Bras. Frutic..

[47] Barboza J.N. (2018). Potencial anti-inflamatório e perfil antioxidante do eugenol: Uma revisão. Trabalho de Conclusão de Curso.

[48] Hýsková V., Ryšlavá H. (2019). Antioxidant Properties of Phenylpropanoids. Biochem Anal. Biochem.

[49] Costa M.D.S., Oliveira-Júnior J.F.D., Santos P.J.D., Correia Filho W.L.F., Gois G.D., Blanco C.J.C., Teodoro P.E., Da Silva C.A., Santiago D.d.B., Souza E.d.O. (2021). Rainfall extremes and drought in Northeast Brazil and its relationship with El Niño–Southern Oscillation. Int. J. Climatol..

[50] Do Socorro M.R.M., Alves R.E., De Brito E.S., Pérez-Jiménez J., Saura-Calixto F., Mancini-Filho J. (2010). Bioactive compounds and antioxidant capacities of 18 non-traditional tropical fruits from Brazil. Food Chem..

[51] Silva E.F.R., Santos B.R.d.S., Brandão G.C., Silva M.V.L., Da Silva E.G.P., Dos Santos W.P.C., Dos Santos W.N.L., Dos Santos A.M.P. (2020). Screening of minerals, proximate composition and physico-chemical characteristics in the discrimination of Oiti (*Licania tomentosa* (Benth.) Fritsch.) using Kohonen self-organizing maps, PCA and HCA. Braz. J. Dev..

